# Surgery

**Published:** 1921-06

**Authors:** James Swain


### SURGERY.

The **treatment of retention of urine in paraplegic patients** is of great importance, for a fatal infection of the urinary tract is a frequent complication of cord injuries. There is no doubt that the former method of treating these cases by catheterism was mainly responsible for the introduction of the organisms which caused such disastrous results.

The subject is discussed[1] by Dr. V. C. David, who established various experimental conditions in dogs in order to study the relation of infection in the urinary tract to cord lesions. His summary of the different methods of treatment is as follows: (1) Catheterism sooner or later causes cystitis, with subsequent ascending infection of the urinary tract. (2) The emptying of the distended bladder by manual expression is a safe method of procedure, as the dangers of catheterism are avoided. On the other hand, the bladder is not completely emptied by this procedure, and the residual urine may become infected from the blood stream. (3) The periodic automatic expulsion of urine by reflex action is an ideal condition, but unfortunately the automatic bladder emptying does not begin until about the twenty-fifth day after injury, and only then under favourable conditions, such as the absence of cystitis. It appears that this automatic action of the bladder is best established by the regular use of the catheter, but this involves the risks to which reference has already been made. (4) Continuous drainage of the bladder by a retained catheter or supra-pubic cystostomy. The former is impracticable owing to the difficulty of maintenance and the trophic disturbance likely to result from pressure on the neck of the bladder and in the urethra. Cystostomy is associated with an absence of residual urine, and prevents dilatation of the bladder, but has the disadvantages of the necessity of operative interference, increased difficulty of nursing, and the certainty of bladder infection.

In estimating the value of these different methods of treatment Dr. David considers that in early cases where there is no urinary infection catheterism is absolutely contra-indicated, and that the distended bladder should be relieved by manual expression of the urine. If this is successful it may later on lead to the establishment of the periodic automatic emptying of the bladder, which may, however, ultimately be established in cases in which manual expression fails.

As long as infection of the urinary tract does not occur manual expression of the urine or periodic reflex urination should be favoured indefinitely. If infection of the residual urine takes place supra-pubic cystostomy and constant drainage of the bladder by siphonage is the procedure to be employed.

**Carcinoma of the Prostate** is considered[2] by Dr. H. C. Bumpus largely from the point of view of the occurrence of metastasis. Very few cases of metastasis from a primary prostatic growth have been reported, but its occurrence is of considerable importance, especially in association with the customary treatment of malignant disease of the prostate by means of radium. That it may be present without giving rise to obvious symptoms, and when the prostate gland is not necessarily enlarged to any marked degree, are facts which Dr. Bumpus specially emphasises, because the results and deductions reported of any form of therapy in prostatic carcinoma are apt to be misleading unless the presence of metastasis has been excluded by means of skiagraphy and neurological examination. His remarks are based on a study of 362 cases of carcinoma of the prostate observed at the Mayo Clinic from 1914 to 1919 inclusive. Of these 79 (21 per cent.) showed evidence of metastasis.

Cancer of the prostate may be primary in the gland, but more frequently it is associated with simple hyperplasia. Two obvious clinical types are described, but many intermediate types occur. In the first type there are few local symptoms, and the gland is only slightly enlarged, with a uniform contour and an absence, generally, of the characteristic hard areas. It is apt to be confused with a simple hypertrophy, but it is wanting in the elasticity which is felt in a simple enlargement. The gland is never so large that it cannot be completely outlined by digital examination. In these cases it not infrequently happens that the occurrence of metastasis leads to the discovery of the primary disease. In the second and more common type the gland may present any degree of enlargement, but the contour is always felt to be irregular and the surface is elevated by areas of stony hardness. Later on these areas coalesce, and the entire gland then feels irregular and very hard.

Metastasis was found in the bones of 41 of the patients who were X-rayed, representing 51 per cent. of the 79 patients with metastasis and 30.3 per cent. of the 135 patients who were examined by X-rays. The pelvis, spine and femur are the most common sites of metastasis in bone.

It is noteworthy that macroscopic hæmaturia and retention of urine as first symptoms were found to be very uncommon in these cases, especially in those associated with metastasis. In the later stages retention occurs in 25 per cent. of the cases with metastasis, and in 33.9 per cent. of the cases without metastasis. The author's conclusions are as follows: (1) Metastasis occurs in the glands more frequently in cases of carcinoma of the prostate than is demonstrable clinically because of the inaccessibility of the glands first involved. (2) Two distinct types of carcinoma of the prostate are distinguishable. (3) The smaller type gives clinical and microscopic evidence of greater malignancy. (4) The larger type tends to remain localised, resulting in more urinary symptoms and producing metastasis later in the disease than the smaller clinical type. (5) The larger clinical type is more amenable to radium therapy because of its lesser potentiality of malignancy. (6) One-third of the patients with carcinoma of the prostate have osseous metastasis demonstrable by X-rays. (7) The pelvis and spine are the most frequent sites of osseous metastasis. (8) Metastasis rarely occurs in the lungs, and probably never without involvement elsewhere. (9) Metastasis in the spinal cord closely simulates primary cord tumours, and often occurs when the cancerous prostate is but slightly enlarged. (10) Pain is absent in one-fourth of all cases with metastasis. (11) Urinary symptoms are absent in 11.5 per cent. of all cases with metastasis. (12) Neuralgic and rheumatic pains in men above middle age, even in the absence of urinary symptoms, should suggest the possibility of carcinoma of the prostate.

**The treatment of perforating gastric and duodenal ulcers** by cœliotomy and suture of the opening is agreed upon by all; but the question as to whether this simple operation should be immediately followed by a gastro-enterostomy or not has been fiercely debated: On the one hand we have those who consider that suture alone is sufficient, and on the other hand we have those who deny that this is so, and insist on the desirability of performing a gastro-enterostomy in all cases where the general condition of the patient is good enough for the purpose. The conservative view is well expressed by Dr. C. F. Farr,[3] who points out that in spite of the argument for gastro-enterostomy that it aids the healing of the ulcer and obviates a later operation for stenosis, it is well known that by far the greater number of perforating ulcers heal well after suture and that hemorrhage and stenosis are exceptional sequelæ.

He considers that the strongest arguments against the added operation are: (1) There is an additional immediate mortality; (2) the end-results of gastro-enterostomy are not 100 per cent. good. It seems wiser to perform only the simple life-saving operation and await the result, and undertake a gastro-enterostomy later on for the small number of cases requiring it.

The other point of view is expressed by Dr. R. Lewisohn,[4] who maintains that it is not uncommon for pyloric and duodenal ulcers to persist after simple suture of an acute perforation. His conclusions are: (1) Immediate gastro-enterostomy in the treatment of perforation does not increase the mortality. (2) Gastro-enterostomy and pyloric exclusion simplify the post-operative treatment. (3) Gastro-enterostomy will guarantee proper drainage of the stomach contents and overcome partial obstruction of the pylorus caused by post-operative adhesions. (4) Closure of the perforation, gastro-enterostomy, and pyloric exclusion should be the method of choice. Simple closure of the perforation should be reserved for those patients whose general condition is so poor that even a rapidly performed gastro-enterostomy would be too much of an operative risk.

So much depends upon the judgment and operative ability of the individual surgeon that it is a difficult matter to decide upon this vexed question. The accumulation in the future of more evidence from the accurate case histories of the two methods of procedure may allow of a just conclusion; but meanwhile it seems desirable that at least surgeons of limited experience should be content with simple suture, which saves the patient from the possibility of a mistake as to his powers of sustaining a more prolonged operation.

REFERENCES.

[1] *J. Am. M. Ass.*, 1921, lxxvi. 494.
[2] *Surg. Gynec. and Obst.*, 1921, xxxii. 31.
[3] *Ann. Surg.*, 1920, lxxii. 591.
[4] *Ibid.*, p. 595.

JAMES SWAIN.

